# Differential analysis for high density tiling microarray data

**DOI:** 10.1186/1471-2105-8-359

**Published:** 2007-09-24

**Authors:** Srinka Ghosh, Heather A Hirsch, Edward A Sekinger, Philipp Kapranov, Kevin Struhl, Thomas R Gingeras

**Affiliations:** 1 Affymetrix Inc., Santa Clara, CA 95051, USA; 2 Dept. Biological Chemistry & Molecular Pharmacology, Harvard Medical School, Boston, MA 02115, USA; 3 Asuragen, Inc., 2150 Woodward, Austin, TX 78744, USA

## Abstract

**Background:**

High density oligonucleotide tiling arrays are an effective and powerful platform for conducting unbiased genome-wide studies. The *ab initio *probe selection method employed in tiling arrays is unbiased, and thus ensures consistent sampling across coding and non-coding regions of the genome. These arrays are being increasingly used to study the associated processes of transcription, transcription factor binding, chromatin structure and their association. Studies of differential expression and/or regulation provide critical insight into the mechanics of transcription and regulation that occurs during the developmental program of a cell. The time-course experiment, which comprises an *in-vivo *system and the proposed analyses, is used to determine if annotated and un-annotated portions of genome manifest coordinated differential response to the induced developmental program.

**Results:**

We have proposed a novel approach, based on a piece-wise function – to analyze genome-wide differential response. This enables segmentation of the response based on protein-coding and non-coding regions; for genes the methodology also partitions differential response with a 5' versus 3' versus intra-genic bias.

**Conclusion:**

The algorithm built upon the framework of Significance Analysis of Microarrays, uses a generalized logic to define regions/patterns of coordinated differential change. By not adhering to the gene-centric paradigm, discordant differential expression patterns between exons and introns have been identified at a FDR of less than 12 percent. A co-localization of differential binding between RNA Polymerase II and tetra-acetylated histone has been quantified at a p-value < 0.003; it is most significant at the 5' end of genes, at a p-value < 10^-13^. The prototype R code has been made available as supplementary material [see Additional file 1].

## Background

Use of DNA microarrays has become commonplace for monitoring the expression levels of thousands of genes simultaneously [1]. The gene expression signature represents the steady state level of RNA in cells and can be utilized to detect cellular response to an exogenous stimulation originating from a treatment, disease or other sources [2-4]. In understanding the dynamics of transcriptional regulation it is imperative to both identify and quantify the response of the loci manifesting differential changes in a comprehensive, genome-wide manner. This requires an exhaustive probing of both the protein coding and non-coding regions of the genome. Tiling array technology has facilitated unbiased genome-wide interrogation. The subsequent challenge is one of bioinformatics, requiring statistical interpretation of voluminous data with potentially low signal to noise ratio (*SNR*) to detect, characterize and quantify differential regulation. In response to this challenge we have proposed generalized SAM (*gSAM*), an extension to the methodology which forms the basis of Significance Analysis of Microarrays (*SAM*) [5].

### The analytical paradigm

Classically, a 2x fold change (*FC*) in gene expression level has been a surrogate for establishing differential change. Regions of the genome with reduced coding potential might not exhibit such FCs. In fact the stringency of the 2x requirement can introduce a strong false negative bias. A more direct approach is to determine if the FCs are significantly different from zero. Hence the null hypothesis (*H*_*0*_) for differential expression/modification is that there is no change in the mean response (*μ*) of a locus due to a change in its condition from *A *to *B *(Eqn. 1). The p-value is simply the probability that FC values drawn from such a distribution are reproducible. Therefore, a low p-value (<0.05) implies that is it highly unlikely that the measured differential response is a consequence of random chance alone. The Student t-test is a classical parametric test used to assign the significance levels (Eqn. 2).

H0=E(μ¯B−μ¯A)=0
 MathType@MTEF@5@5@+=feaafiart1ev1aaatCvAUfKttLearuWrP9MDH5MBPbIqV92AaeXatLxBI9gBaebbnrfifHhDYfgasaacH8akY=wiFfYdH8Gipec8Eeeu0xXdbba9frFj0=OqFfea0dXdd9vqai=hGuQ8kuc9pgc9s8qqaq=dirpe0xb9q8qiLsFr0=vr0=vr0dc8meaabaqaciaacaGaaeqabaqabeGadaaakeaacqWGibasdaWgaaWcbaGaeGimaadabeaakiabg2da9iabdweafjabcIcaOGGaciqb=X7aTzaaraWaaSbaaSqaaiabdkeacbqabaGccqGHsislcuWF8oqBgaqeamaaBaaaleaacqWGbbqqaeqaaOGaeiykaKIaeyypa0JaeGimaadaaa@3BB7@

t−statistic=((μ¯B−μ¯A)−0σ^(μ¯B−μ¯A))
 MathType@MTEF@5@5@+=feaafiart1ev1aaatCvAUfKttLearuWrP9MDH5MBPbIqV92AaeXatLxBI9gBaebbnrfifHhDYfgasaacH8akY=wiFfYdH8Gipec8Eeeu0xXdbba9frFj0=OqFfea0dXdd9vqai=hGuQ8kuc9pgc9s8qqaq=dirpe0xb9q8qiLsFr0=vr0=vr0dc8meaabaqaciaacaGaaeqabaqabeGadaaakeaacqWG0baDcqGHsislcqWGZbWCcqWG0baDcqWGHbqycqWG0baDcqWGPbqAcqWGZbWCcqWG0baDcqWGPbqAcqWGJbWycqGH9aqpdaqadaqaamaalaaabaGaeiikaGccciGaf8hVd0MbaebadaWgaaWcbaGaemOqaieabeaakiabgkHiTiqb=X7aTzaaraWaaSbaaSqaaiabdgeabbqabaGccqGGPaqkcqGHsislcqaIWaamaeaacuWFdpWCgaqcaiabcIcaOiqb=X7aTzaaraWaaSbaaSqaaiabdkeacbqabaGccqGHsislcuWF8oqBgaqeamaaBaaaleaacqWGbbqqaeqaaOGaeiykaKcaaaGaayjkaiaawMcaaaaa@5349@

There are obvious deficiencies in this analytical paradigm; the primary one arises from the fact that microarray data follows a non-normal distribution [6]. It can be argued that the t-test results remain asymptotically correct for any distribution but only if the number of replicates tend to infinity. This makes an experiment logistically difficult and cost-prohibitive. Thus, in a global sense, due to the inaccurate definition of H_0 _the classical approach does not verify if the genes are truly differentially regulated or are false positives of a stochastic origin.

Multiple hypothesis testing is the other element that needs to be addressed. Table 1 recounts its fundamental principles and the error rates as summarized in Benjamini and Hochberg [7]; the following summary of error rates utilizes the symbols defined in the table. Fundamentally, there are two types of error rates [7-11]: type I or false positive (M_0_-F) and type II or false negative (T); the former is associated with rejection of a true null hypothesis and the latter with the failure to reject the false null hypothesis. For microarray experiments, control of the type I error under any combination of the true and false hypotheses is critical [11]. Briefly, the type I error rates are:

**Table 1:** Multiple hypothesis testing matrix

	**Hypotheses: Accepted**	**Hypotheses: Rejected**	**Total**
Null: True (Null: no differential change)	F	M0 – F	M0
Alternative: True Or Null: False	T	M1 – T	M1
Total	S	M – S	M

i) *Per family error rate *(*PFER*): refers to the expected number of false positives (Eqn. 3);

ii) *Per comparison error rate *(*PCER*): refers to the expected value of the number of false positives compared to the number of hypotheses (Eqn. 4);

iii) *Family-wise error rate *(*FWER*): refers to the probability of at least one false positive [7,12-14] (Eqn. 5);

iv) *False Discovery Rate *(*FDR*): refers to the expected proportion of false positives among rejected hypotheses [7,12,15,16] (Eqn. 6);

*PFER *= *E*(*M*_0 _- *F*)

PCER=E(M0−F)M
 MathType@MTEF@5@5@+=feaafiart1ev1aaatCvAUfKttLearuWrP9MDH5MBPbIqV92AaeXatLxBI9gBaebbnrfifHhDYfgasaacH8akY=wiFfYdH8Gipec8Eeeu0xXdbba9frFj0=OqFfea0dXdd9vqai=hGuQ8kuc9pgc9s8qqaq=dirpe0xb9q8qiLsFr0=vr0=vr0dc8meaabaqaciaacaGaaeqabaqabeGadaaakeaacqWGqbaucqWGdbWqcqWGfbqrcqWGsbGucqGH9aqpdaWcaaqaaiabdweafjabcIcaOiabd2eannaaBaaaleaacqaIWaamaeqaaOGaeyOeI0IaemOrayKaeiykaKcabaGaemyta0eaaaaa@3A6B@

*FWER *= *p*((*M*_0 _- *F*) > 0)

FDR=E((M0−F)(M−S)) if (M−S)>0
 MathType@MTEF@5@5@+=feaafiart1ev1aaatCvAUfKttLearuWrP9MDH5MBPbIqV92AaeXatLxBI9gBaebbnrfifHhDYfgasaacH8akY=wiFfYdH8Gipec8Eeeu0xXdbba9frFj0=OqFfea0dXdd9vqai=hGuQ8kuc9pgc9s8qqaq=dirpe0xb9q8qiLsFr0=vr0=vr0dc8meaabaqaciaacaGaaeqabaqabeGadaaakeaacqWGgbGrcqWGebarcqWGsbGucqGH9aqpcqWGfbqrdaqadaqaamaalaaabaGaeiikaGIaemyta00aaSbaaSqaaiabicdaWaqabaGccqGHsislcqWGgbGrcqGGPaqkaeaacqGGOaakcqWGnbqtcqGHsislcqWGtbWucqGGPaqkaaaacaGLOaGaayzkaaGaeeiiaaIaemyAaKMaemOzayMaeeiiaaIaeiikaGIaemyta0KaeyOeI0Iaem4uamLaeiykaKIaeyOpa4JaeGimaadaaa@49C2@

In general, the procedures controlling the FWER are more conservative than the ones controlling the PCER or FDR. Hence the classical Bonferroni correction (FWER) is much too stringent for array-based differential regulation studies, especially encompassing partially coding to non-coding regions. The SAM algorithm, built on a re-sampling framework, virtually, increases the number of replicates, via random permutation of the sample labels; this formalizes a refinement to the multiple-testing corrected p-value and false positive rate (*FPR*) and is referred to as the q-value and FDR [5,17-19]. Fundamentally, the test statistic in SAM (Eqn. 7) is a t-statistic variant where a constant (*s*_*0*_) is added to the variance term in the denominator. s_0_, computed empirically controls for a reduction in SNR with decreasing differential change. Traditionally, the d-statistic is defined as a function of a *gene *under two conditions *A *and *B*, but in gSAM this has been generalized to a genomic interval, *I*.

d−statistic(I)=((μ¯B(I)−μ¯A(I))s(I)−s0)
 MathType@MTEF@5@5@+=feaafiart1ev1aaatCvAUfKttLearuWrP9MDH5MBPbIqV92AaeXatLxBI9gBaebbnrfifHhDYfgasaacH8akY=wiFfYdH8Gipec8Eeeu0xXdbba9frFj0=OqFfea0dXdd9vqai=hGuQ8kuc9pgc9s8qqaq=dirpe0xb9q8qiLsFr0=vr0=vr0dc8meaabaqaciaacaGaaeqabaqabeGadaaakeaacqWGKbazcqGHsislcqWGZbWCcqWG0baDcqWGHbqycqWG0baDcqWGPbqAcqWGZbWCcqWG0baDcqWGPbqAcqWGJbWycqGGOaakcqWGjbqscqGGPaqkcqGH9aqpdaqadaqaamaalaaabaGaeiikaGccciGaf8hVd0MbaebadaWgaaWcbaGaemOqaieabeaakiabcIcaOiabdMeajjabcMcaPiabgkHiTiqb=X7aTzaaraWaaSbaaSqaaiabdgeabbqabaGccqGGOaakcqWGjbqscqGGPaqkcqGGPaqkaeaacqWGZbWCcqGGOaakcqWGjbqscqGGPaqkcqGHsislcqWGZbWCdaWgaaWcbaGaeGimaadabeaaaaaakiaawIcacaGLPaaaaaa@56EE@

### Basics of gSAM

The purpose of gSAM is to transform genomic intervals of enrichment originating from changes in RNA levels, binding/occupancy of transcriptional regulators, modified histones, levels of chromatin modification, among others, to a temporal/spatial differential signature for these elements. Unlike gene-centric expression arrays which have a 3' end bias or exon arrays which specifically interrogate the exons, in tiling arrays multiple probes interrogate a single locus in an unbiased manner. Here a locus can encompass multiple transcripts and/or interaction sites of multiple regulatory elements and can include exons, introns and un-translated regions (*UTRs*). Therefore, instead of computing a gene-level (with 3' bias) differential measure, in gSAM the differential measurement follows a piece-wise response model. This is described in Eqn. 8 where *ig, ex, in, UTR *correspond to the inter-genic, exon, intron and un-translated region respectively. Under this model, the time-series, for example, is subdivided into a number of *logical *segments – in this case the underlying logic is governed by enrichment – and differential change is summarized over each segment. Fundamentally, the definition of the segments is completely independent of annotations. This enables extension of the methodology to beyond the framework of annotations and hence to those genomes other than human where the annotation is not as complete. However, the availability of annotation facilitates visualization of the outcome from a protein-coding perspective.

The piece-wise system model in gSAM supports two inherent characteristics of transcriptome data – heterogeneity and superposition of states. This is demonstrated in Eqn. 9 where, for example, the inter-genic component is a superposition of states with *n *variable enrichment patterns. According to current knowledge, SAM assumes a homogenous and static one-gene, one-locus model; the implicit assumption being that differential response is not a complex, superposition of responses but is a homogenous/uniform response across all nucleotides comprising a gene. Consideration of a gene as an atomic entity does not enable discrimination of the differential response of alternative isoforms in a developmental transcriptome or even exons versus introns versus UTRs for a transcript. The *system *definition which is the primary point of differentiation between SAM and gSAM consequently impacts the interpretation of the differential changes at a cellular level. The following sections elucidate the rationale underlying gSAM and discuss its impact on transcriptome-level differential data analysis.

*f*(Δ)_*A*,*B *_→ (*f*(*ig*) + *f*(*ex*) + *f*(*in*) + *f*(*UTR*))_*A,B*_

*f*(*ig*) → *χ*(*ig*)_1 _+ ... + *χ*(*ig*)_*n*_

## Methods

### Time course experimental design

The development and application of gSAM are presented here in the context of a differential time-course study conducted in HL60 cell-line, performed as part of the Encyclopedia of DNA elements (*ENCODE*) consortium project [20-22]. The cells are stimulated by all-trans retinoic acid (*ATRA*) for distinct time periods – 0, 2, 8 and 32 hours – to induce differentiation along the granulocytic lineage. The biological motivation of the experiments is to study the associated processes of RNA transcription, the binding of transcriptional regulators, and to identify regions of histone modification. The differential RNA transcription [23-25] comprises a single sample experiment where the level of RNA is monitored with respect to a baseline as quantified via negative control probes based on bacterial sequences. The differential modification study involves a two-sample chromatin immunoprecipitation on array/chip (*ChIP on chip*) experiment [26-34] comprising a control and treatment. The control is amplified genomic DNA (without immunoprecipitation), and the treatment is the chromatin immunoprecipitated sample. The assay protocol used in these experiments is not strand specific; this is a method of sample preparation that does not preserve information about the strand of the nucleic acids, hence it cannot be discerned conclusively as to which strand the observed effects originate from. An example of such method is conversion of RNA into double-stranded cDNA (used in these experiments) for measuring RNA abundance. Details regarding the specific assays have been described in the literature [24,34]. The example biological datasets used to demonstrate the application of gSAM include RNA (whole-cell poly A+), a trio of modified histones: *H4Kac4*-Histone H4 tetra-acetylated lysine (*HisH4*), *H3K9K14ac2 *-Histone H3 K9 K14 di-acetylated (*H3K9K14D*), *H3K27me3*-Histone H3 tri-methylated lysine 27(*H3K27T*) and *RNA Polymerase II-*8WG16 antibody against pre-initiation complex form (*RNA PolII*). For each regulation factor investigated, the experiment comprises three to five biological replicates, per time-point, with duplicate hybridizations performed for each.

### Tiling arrays – the Affymetrix platform

These arrays employ short oligonucleotide probe-pairs (*pp*), of length 25 bases (25 mers), to interrogate a specified genomic region [35-37]. Each pp includes a perfect match (*PM*) and a mismatch (*MM*). The MM sequence is identical to its corresponding PM sequence, except for the central (13^th^) base. The objective of pairing a PM with a MM is to estimate the degree of cross-hybridization. A variety of tiling arrays with different probe and feature resolution are used for genome-wide transcription regulation studies [38-40]. The probe resolution defines the center to center distance between two adjacent probes, in genomic space. A 22 base-pair (*bp*) probe resolution for 25 mers implies a 3 bp overlap (on average) between 2 adjacent probes. Currently, the probe resolution of the arrays encompasses a range from 5 bp-35 bp with probe synthesis areas of 5*μ *and 10*μ*.

### Application of gSAM for detection of differential change

gSAM operates on enrichment site-level data and estimates the temporal differential regulation signature. The H_0 _in this study is that there is no difference in RNA levels, histone modification or binding of regulators due to stimulation by ATRA over a designated time-course. Although the methodology encompasses both PM and MM probes, it can be extended to PM only arrays or exclude MM probes. The following sections detail the algorithmic steps:

I. Preliminary data analysis

II. Definition of the pair-wise system

III. Modeling the input to gSAM

IV. Probe-level signal intensity/enrichment summarization

V. Summarization of differential response

#### I. Preliminary data analysis

This section summarizes the steps for the generation of sites corresponding to RNA or modified histone and/or RNA PolII binding.

i) Probe-level normalization: This includes median scaling and quantile normalization [41,42] of all PM and MM probes. The former is a linear operation, where fluorescence data from the arrays are scaled relative to the median intensity distributions of all arrays. The quantile normalization accounts for linear and non-linear effects.

ii) RNA profiling experiments: The pp signal intensity (*SI*) distribution is computed based on PM-MM intensity; regions of detected RNA referred to as transfrags (transcribed fragments) are then estimated against a baseline transcription signal derived from both positive and negative bacterial controls on the same microarray. For the data presented here, the intensity threshold for transcriptionally positive probes is set based on a 5 percent FPR [23-25].

iii) ChIP on chip experiments: The probe-level signal enrichment (*SE*) profiles are generated based on a comparison of the signal intensity of the treatment and control probe pairs (Eqn. 10). Putative transcriptional regulatory elements (*TREs*) are generated per factor on a per time point basis using the Rank Statistics based site prediction algorithm [43]. In general, the enriched fragments exhibit the following types of bias [31]:

a) Canonical regulatory sites have a 5'end bias;

b) Non-canonical sites are distal to the annotated 5'ends[22,31,44];

SEpp=max⁡(1,log⁡(PM−MM)Treatment)ppmax⁡(1,log⁡(PM−MM)Control)pp
 MathType@MTEF@5@5@+=feaafiart1ev1aaatCvAUfKttLearuWrP9MDH5MBPbIqV92AaeXatLxBI9gBaebbnrfifHhDYfgasaacH8akY=wiFfYdH8Gipec8Eeeu0xXdbba9frFj0=OqFfea0dXdd9vqai=hGuQ8kuc9pgc9s8qqaq=dirpe0xb9q8qiLsFr0=vr0=vr0dc8meaabaqaciaacaGaaeqabaqabeGadaaakeaacqWGtbWucqWGfbqrdaWgaaWcbaGaemiCaaNaemiCaahabeaakiabg2da9maalaaabaGagiyBa0MaeiyyaeMaeiiEaGNaeiikaGIaeGymaeJaeiilaWIagiiBaWMaei4Ba8Maei4zaCMaeiikaGIaemiuaaLaemyta0KaeyOeI0Iaemyta0Kaemyta0KaeiykaKYaaSbaaSqaaiabdsfaujabdkhaYjabdwgaLjabdggaHjabdsha0jabd2gaTjabdwgaLjabd6gaUjabdsha0bqabaGccqGGPaqkdaWgaaWcbaGaemiCaaNaemiCaahabeaaaOqaaiGbc2gaTjabcggaHjabcIha4jabcIcaOiabigdaXiabcYcaSiGbcYgaSjabc+gaVjabcEgaNjabcIcaOiabdcfaqjabd2eanjabgkHiTiabd2eanjabd2eanjabcMcaPmaaBaaaleaacqWGdbWqcqWGVbWBcqWGUbGBcqWG0baDcqWGYbGCcqWGVbWBcqWGSbaBaeqaaOGaeiykaKYaaSbaaSqaaiabdchaWjabdchaWbqabaaaaaaa@7526@

#### II. Definition of the pair-wise system

This section provides a rationale for the choice of pair-wise conditions at which the cellular responses are profiled and analyzed.

Cellular response to an exogenous stimulus is not necessarily synchronized; however the reaction is on a very short time-scale – essentially continuous. In capturing events over time-points separated on the order of hours, a discrete time-differential response is generated by sampling a continuous time-signal. The sampling process is analogous to an *accumulator system*[45] where the output state of the system (*y*) at any given time *n *is essentially a summation/accumulation of the response of all its states (*x*) up to the present state x[n] (Eqn. 11). Although the superimposed cellular states measured by the experiment cannot be de-convoluted, fundamentally because of the mentioned system characteristic, there is information loss when the states are profiled at large time intervals. Temporal resolution therefore is a critical component of the experimental design. The optimal resolution varies for different responding functional elements, conditions of cell growth and cell/tissue/organism type, with a likelihood of non-linear increments in the time-series. In this particular study, the choice of 0-2-8-32 hours represents the undifferentiated state, an early time point (2 hours), a midway time point (8 hours) and a moderately late time point (32 hours) based on the previously published profiles of HL60 differentiation [46,47].

The associated property that needs to be appreciated is that the differential response follows a *cascade connection *model [45]. Here the un-stimulated(baseline) state at the 0 hour serves as the original input to the system; the output(response) at the 2 hour serves as the input to the 8 hour with the output of the 32 hour (latest) being the overall output. Thus any measurement performed at any state other than the baseline has a memory of the system even prior to its current state.

y[n]=∑t=0nx[t]
 MathType@MTEF@5@5@+=feaafiart1ev1aaatCvAUfKttLearuWrP9MDH5MBPbIqV92AaeXatLxBI9gBaebbnrfifHhDYfgasaacH8akY=wiFfYdH8Gipec8Eeeu0xXdbba9frFj0=OqFfea0dXdd9vqai=hGuQ8kuc9pgc9s8qqaq=dirpe0xb9q8qiLsFr0=vr0=vr0dc8meaabaqaciaacaGaaeqabaqabeGadaaakeaacqWG5bqEcqGGBbWwcqWGUbGBcqGGDbqxcqGH9aqpdaaeWbqaaiabdIha4jabcUfaBjabdsha0jabc2faDbWcbaGaemiDaqNaeyypa0JaeGimaadabaGaemOBa4ganiabggHiLdaaaa@3F88@

Δy=y[T]−y[T−n]=∑t=0Tx[t]−∑t=0n<Tx[t]=∑t=0n<Tx[t]+∑t=n<TTx[t]−∑t=0n<Tx[t]Δy=y[T]−y[T−n]=∑t=n<TTx[t]
 MathType@MTEF@5@5@+=feaafiart1ev1aaatCvAUfKttLearuWrP9MDH5MBPbIqV92AaeXatLxBI9gBaebbnrfifHhDYfgasaacH8akY=wiFfYdH8Gipec8Eeeu0xXdbba9frFj0=OqFfea0dXdd9vqai=hGuQ8kuc9pgc9s8qqaq=dirpe0xb9q8qiLsFr0=vr0=vr0dc8meaabaqaciaacaGaaeqabaqabeGadaaakeaafaqaaeGabaaabaGaeuiLdqKaemyEaKNaeyypa0JaemyEaKNaei4waSLaemivaqLaeiyxa0LaeyOeI0IaemyEaKNaei4waSLaemivaqLaeyOeI0IaemOBa4Maeiyxa0Laeyypa0ZaaabCaeaacqWG4baEcqGGBbWwcqWG0baDcqGGDbqxaSqaaiabdsha0jabg2da9iabicdaWaqaaiabdsfaubqdcqGHris5aOGaeyOeI0YaaabCaeaacqWG4baEcqGGBbWwcqWG0baDcqGGDbqxaSqaaiabdsha0jabg2da9iabicdaWaqaaiabd6gaUjabgYda8iabdsfaubqdcqGHris5aOGaeyypa0ZaaabCaeaacqWG4baEcqGGBbWwcqWG0baDcqGGDbqxaSqaaiabdsha0jabg2da9iabicdaWaqaaiabd6gaUjabgYda8iabdsfaubqdcqGHris5aOGaey4kaSYaaabCaeaacqWG4baEcqGGBbWwcqWG0baDcqGGDbqxaSqaaiabdsha0jabg2da9iabd6gaUjabgYda8iabdsfaubqaaiabdsfaubqdcqGHris5aOGaeyOeI0YaaabCaeaacqWG4baEcqGGBbWwcqWG0baDcqGGDbqxaSqaaiabdsha0jabg2da9iabicdaWaqaaiabd6gaUjabgYda8iabdsfaubqdcqGHris5aaGcbaGaeuiLdqKaemyEaKNaeyypa0JaemyEaKNaei4waSLaemivaqLaeiyxa0LaeyOeI0IaemyEaKNaei4waSLaemivaqLaeyOeI0IaemOBa4Maeiyxa0Laeyypa0ZaaabCaeaacqWG4baEcqGGBbWwcqWG0baDcqGGDbqxaSqaaiabdsha0jabg2da9iabd6gaUjabgYda8iabdsfaubqaaiabdsfaubqdcqGHris5aaaaaaa@ABD3@

These two properties, motivates the quantification of the temporal differential response as a *pairwise time-forward *system encompassing very specific reference and target time-points; Eqn. 12 generalizes this concept. A time-forward analysis implies that samples obtained at (T-n)^th ^and T^th^(where n<T) time-points comprise the reference and target respectively. Here the reference precedes the target time-point, and may or may not represent the un-stimulated condition. While measurement of a response between two time-points might seem trivial, given the underlying accumulator and cascade connection properties, the choice of these time-points is critical; a pairwise combination at random, without appropriate de-convolution will result in erroneous interpretation of the underlying biology. Measurement of first order effects, which is the difference between two contiguous time-points profiled, is simpler to interpret than higher order effects, which include differential profiling across non-contiguous time-points potentially involving non-linear effects.

For the described time-series experiment, a measurement of an increased differential response from 0 to 8 hours, without knowledge of the 2 hour time-point, does not uniquely characterize the underlying differential mechanism. Any of the following are equally probable for a given locus:

i) Between 0 and 8 hours, there is a steady increase in response to ATRA stimulus;

ii) There is an initial decrease in the response between 0 and 2 hours, with a subsequent increase between 2 and 8 hours;

iii) There is a rapid increase in response between 0 and 2 hours with a significantly slower decrease in response between 2 and 8 hours;

Quantification of the first order response slopes significantly reduces the complexity of interpretation. All results presented here comprise the first order differential analysis. Although, gSAM is presented in a temporal context, it is equally applicable in a spatial one; this facilitates quantification of differential response across tissue-types derived from normal (reference) and diseased (target) sites, for example.

#### III. Modeling the input to gSAM

This section summarizes the logical segmentation of the enrichment regions which constitutes the input model for gSAM.

Based on published research [23-25,48-50], presumed non-coding transcripts of yet unknown functionality are widespread in the genome. Thus the analysis of differential response should not be biased toward protein-coding genes but be based on a generalized framework. The generalization in gSAM arises primarily from the piece-wise modeling of the input, which simultaneously accommodates for responses from genic and inter-genic regions.

The gSAM piece-wise model introduced in Eqn. 8–9 is elaborated in Eqn. 13–14. Fragmented enrichment sites – histone/RNA PolII binding sites, transfrags of canonical and/or non-canonical origin, emanating from the coding and/or non-coding regions of the genome, independent of annotation, serve as the input. Eqn. 13 defines the *probe-specific *input, where the atomic entity is a probe-pair; the differential response is estimated individually for each pp encompassing an enrichment site (*ε*).

*Input *= {*pp *| *pp *∈ *ε*}

Input=∑gGenicg+∑igIntergenicigGenic[g]=∑αppα;Intergenic[ig]=∑βppβ; pp∈εGenic={Exon,Intron,UTR}Intergenic={FunctionalComplexity,SequenceComplexity,...}
 MathType@MTEF@5@5@+=feaafiart1ev1aaatCvAUfKttLearuWrP9MDH5MBPbIqV92AaeXatLxBI9gBaebbnrfifHhDYfgasaacH8akY=wiFfYdH8Gipec8Eeeu0xXdbba9frFj0=OqFfea0dXdd9vqai=hGuQ8kuc9pgc9s8qqaq=dirpe0xb9q8qiLsFr0=vr0=vr0dc8meaabaqaciaacaGaaeqabaqabeGadaaakeaafaqaaeabbaaaaeaacqWGjbqscqWGUbGBcqWGWbaCcqWG1bqDcqWG0baDcqGH9aqpdaaeqbqaaiabdEeahjabdwgaLjabd6gaUjabdMgaPjabdogaJnaaBaaaleaacqWGNbWzaeqaaaqaaiabdEgaNbqab0GaeyyeIuoakiabgUcaRmaaqafabaGaemysaKKaemOBa4MaemiDaqNaemyzauMaemOCaiNaem4zaCMaemyzauMaemOBa4MaemyAaKMaem4yam2aaSbaaSqaaiabdMgaPjabdEgaNbqabaaabaGaemyAaKMaem4zaCgabeqdcqGHris5aaGcbaGaem4raCKaemyzauMaemOBa4MaemyAaKMaem4yamMaei4waSLaem4zaCMaeiyxa0Laeyypa0ZaaabuaeaacqWGWbaCcqWGWbaCdaWgaaWcbaacciGae8xSdegabeaaaeaacqWFXoqyaeqaniabggHiLdGccqGG7aWocqWGjbqscqWGUbGBcqWG0baDcqWGLbqzcqWGYbGCcqWGNbWzcqWGLbqzcqWGUbGBcqWGPbqAcqWGJbWycqGGBbWwcqWGPbqAcqWGNbWzcqGGDbqxcqGH9aqpdaaeqbqaaiabdchaWjabdchaWnaaBaaaleaacqWFYoGyaeqaaaqaaiab=j7aIbqab0GaeyyeIuoakiabcUda7iabbccaGiabdchaWjabdchaWjabg2da9iab=v7aLbqaaiabdEeahjabdwgaLjabd6gaUjabdMgaPjabdogaJjabg2da9iabcUha7jabdweafjabdIha4jabd+gaVjabd6gaUjabcYcaSiabdMeajjabd6gaUjabdsha0jabdkhaYjabd+gaVjabd6gaUjabcYcaSiabdwfavjabdsfaujabdkfasjabc2ha9bqaaiabdMeajjabd6gaUjabdsha0jabdwgaLjabdkhaYjabdEgaNjabdwgaLjabd6gaUjabdMgaPjabdogaJjabg2da9iabcUha7jabdAeagjabdwha1jabd6gaUjabdogaJjabdsha0jabdMgaPjabd+gaVjabd6gaUjabdggaHjabdYgaSjabdoeadjabd+gaVjabd2gaTjabdchaWjabdYgaSjabdwgaLjabdIha4jabdMgaPjabdsha0jabdMha5jabcYcaSiabdofatjabdwgaLjabdghaXjabdwha1jabdwgaLjabd6gaUjabdogaJjabdwgaLjabdoeadjabd+gaVjabd2gaTjabdchaWjabdYgaSjabdwgaLjabdIha4jabdMgaPjabdsha0jabdMha5jabcYcaSiabc6caUiabc6caUiabc6caUiabc2ha9baaaaa@F4C3@

Eqn. 14 defines a *probe-set *specific input; here, a probe-set (*α*/*β*), which is a cluster of probe-pairs, interrogates a sequence of nucleotides spanning *ε*. This suggests a heterogeneous model which at the most generalized level is a superposition of genic (*g*) and inter-genic (*ig*) states. The genic state can be further partitioned, based on annotations, into elements such as exons, introns and UTRs. Analysis can be performed independently on each element or on the cumulative elements. The flexibility of selective inclusion of genic elements enhances the localization and specificity of estimation of gene-expression effects in various pathways. For example, this enables the localization of activity in regulatory elements with a 3'UTR bias [51]. A response aggregated over the entirety of the genic components would not elucidate this. The inter-genic state is a mixture model as well, encompassing variants in terms of functional and sequence complexity. It can be partitioned based on regulatory potential, for example presence of CpG islands associated with gene expression, regions of sequence conservation, or sequence motifs for transcription factor(s). The framework to selectively integrate elements in the model, solely driven by co-regulation effects, highlights the adaptability and power of gSAM.

#### IV. Probe-level signal intensity/enrichment summarization

This section details the probe-specific summarization of signal intensity/enrichment, for pair-wise sample (*s*).

Subsequent to the setup of the gSAM model, a probe-specific or probe-set specific log transformed SI or SE is computed per replicate(*r *∈ *s*) and time-point (*t *∈ *s*). This constitutes the input value in gSAM. For a probe-set based system, the transfrags/binding sites are used to define the *domain *over which the intensity/enrichment summary is computed. Frequently, the enrichment sites as determined in the reference and target pairs have non-identical spatial bounds. This might be because of biological reasons: the same locus might not be enriched (expressed) at two different time-points; alternatively, this might be due to edge artifacts in the definition of enrichment sites [43], or a combination of both. This incongruity of bounds requires a formal definition of domains based on the segmentation of enriched fragments that are unique to, versus common in both the reference and target samples. Once the domains are defined for a pair-wise sample, they are held constant across all replicates in the relevant reference and target. The following describes the definition of domains and estimation of domain-specific summaries:

i) The first step involves outlier removal. The low complexity filter (*LCF*) estimates the median absolute deviation (*MAD*) of the SE/SI of all pp belonging to a fragment. All fragments with a MAD value of zero are assumed to represent signal from low complexity repeat probes and are therefore eliminated. It is possible that this step introduces a false negative bias, by eliminating enriched fragments composed of a shorter run of probes; this is not detrimental, since the statistical confidence obtained from less than three contiguous probes(66 nucleotides) – is low (data not shown). Independent of LCF enriched fragments with a minimum of three probe-sets is retained. Since these filters address a tiling design and/or sequence specific properties, their effect is assumed to be equivalent for all replicates and is therefore assessed based on a single replicate. Additionally, the MAD serves as a metric of co-regulation. Based on the cumulative MAD distribution, as estimated across all enriched fragments, a user defined threshold can be determined and only fragments with less than the MAD cutoff can be retained for analysis. This filter is replicate quality dependent and should be used with caution, since it can introduce incongruity of bounds.

ii) In this step, the enriched fragments are ordered and labeled – independently in the reference and target – based on their genomic location. It is probable that the bounds of a single enrichment site in the reference might overlap with multiple sites in the target (or vice-versa). In this case, the single reference site (R) has *n *associated target labels, where n corresponds to the number of distinct target sites (T_1_...T_n_) it overlaps with. This labeling scheme identifies the membership of fragments and their relationship across the reference and target.

iii) This step entails identification of genomic segments with overlapping (including partial overlaps) spatial bounds of enrichment between the reference and target. A union of the bounds of the overlapping regions is created. This is referred to as the *overlapping enrichment domain *(*OED*) distribution for a given sample(*s*) (Eqn. 15). The OED therefore comprises a mixture of enriched segments: a common enrichment fraction between reference and target, and a unique fraction with evidence of enrichment in either the reference or target.

iv) In a given OED, the probes interrogating the intersecting and unique enrichment fractions comprise the *FragmentDomainI (FDI) *and *FragmentDomainU (FDU)*, respectively (Eqn. 17–18).

v) This step localizes genomic segments with non-overlapping bounds of enrichment between the reference and target. By definition, these segments have no enriched probes in their counterpart samples and these are referred to as *null *probes. This is referred to as the *non-overlapping enrichment domain *(*NOED*) (Eqn. 18).

vi) Subsequent to the segmentation, there exist three distinct types of enrichment domains: FDI, FDU and NOED. Elements of each domain are denoted by start and stop coordinates to specify their bounds (Eqn. 15) and their reference and target specific labels. For a pair-wise analysis these domains are uniquely labeled and ordered based on their genomic location. A comparison of the labels across the domains – FDI and FDU – potentially identifies differential response from alternative isoforms and/or provides a tool to isolate differential signal from selective genic elements, for example UTRs. Since the enrichment sites are generated in the first-place via a multi-replicate analysis, the spatial bounds of the above domains are held constant across all replicates within a given reference or target.

*OED*_*s *_= (*Enrichment*_*R *_⋃ *Enrichment*_*T*_)_*s *_*where Enrichment*_*R *_∩ *Enrichment*_*T *_> 0

*FDI*_*s *_= (*Enrichment*_*R *_⋂ *Enrichment*_*T*_)_*s *_*where FDI*_*s *_⊂ *OED*_*s*_

*FDU*_*s *_= *OED*_*s *_- *FDI*_*s*_

*NOED*_*s *_= (*Enrichment*_*R *_∩ *Enrichment*_*T *_= Φ)_*s *_*where *Φ : *nullset*

vii) RNA transcription: The gSAM test-statistic operates on the log transformed probe-pair signal intensity (*SI*_*pp*_). For a domain-specific input, a trimmed mean signal intensity(*TRSI*_*drt*_) estimate (Eqn. 19) is generated for each of the domain elements on a per replicate(*r*), per time-point (*t*) basis. This estimate considers all probes belonging to an element in a given domain and uses an optimal trim factor of *κ *= 0.2.

viii) ChIP on chip: gSAM operates on the winsorized mean (robust estimator) (Eqn. 20) of the SE of all probe-pairs per element per labeled domain; these estimates are also computed per replicate and per time-point. In Eqn. 20, *n *refers to the number of probe-pairs in an element of a given domain and *k *refers to the number of smallest and largest observations that are replaced with (k+1)^th ^smallest and largest observations respectively.

ix) The *null *probes in NOED are not set to zero, but their true signal intensities considered. This obviates the missing data problem in gSAM.

*TRSI*_*drt *_= *TrimmedMean*((log(*SI*_1_...*SI*_*pp*_)),0.2)_*drt*_

x¯k=1(n−2k)∑i=k+1n−kxixw=kx(k+1)+x¯k(n−2k)+kx(n−k)n
 MathType@MTEF@5@5@+=feaafiart1ev1aaatCvAUfKttLearuWrP9MDH5MBPbIqV92AaeXatLxBI9gBaebbnrfifHhDYfgasaacH8akY=wiFfYdH8Gipec8Eeeu0xXdbba9frFj0=OqFfea0dXdd9vqai=hGuQ8kuc9pgc9s8qqaq=dirpe0xb9q8qiLsFr0=vr0=vr0dc8meaabaqaciaacaGaaeqabaqabeGadaaakeaafaqadeGabaaabaGafmiEaGNbaebadaWgaaWcbaGaem4AaSgabeaakiabg2da9maalaaabaGaeGymaedabaGaeiikaGIaemOBa4MaeyOeI0IaeGOmaiJaem4AaSMaeiykaKcaamaaqahabaGaemiEaG3aaSbaaSqaaiabdMgaPbqabaaabaGaemyAaKMaeyypa0Jaem4AaSMaey4kaSIaeGymaedabaGaemOBa4MaeyOeI0Iaem4AaSganiabggHiLdaakeaacqWG4baEdaWgaaWcbaGaem4DaChabeaakiabg2da9maalaaabaGaem4AaSMaemiEaG3aaSbaaSqaaiabcIcaOiabdUgaRjabgUcaRiabigdaXiabcMcaPaqabaGccqGHRaWkcuWG4baEgaqeamaaBaaaleaacqWGRbWAaeqaaOGaeiikaGIaemOBa4MaeyOeI0IaeGOmaiJaem4AaSMaeiykaKIaey4kaSIaem4AaSMaemiEaG3aaSbaaSqaaiabcIcaOiabd6gaUjabgkHiTiabdUgaRjabcMcaPaqabaaakeaacqWGUbGBaaaaaaaa@6802@

**Figure 1:**
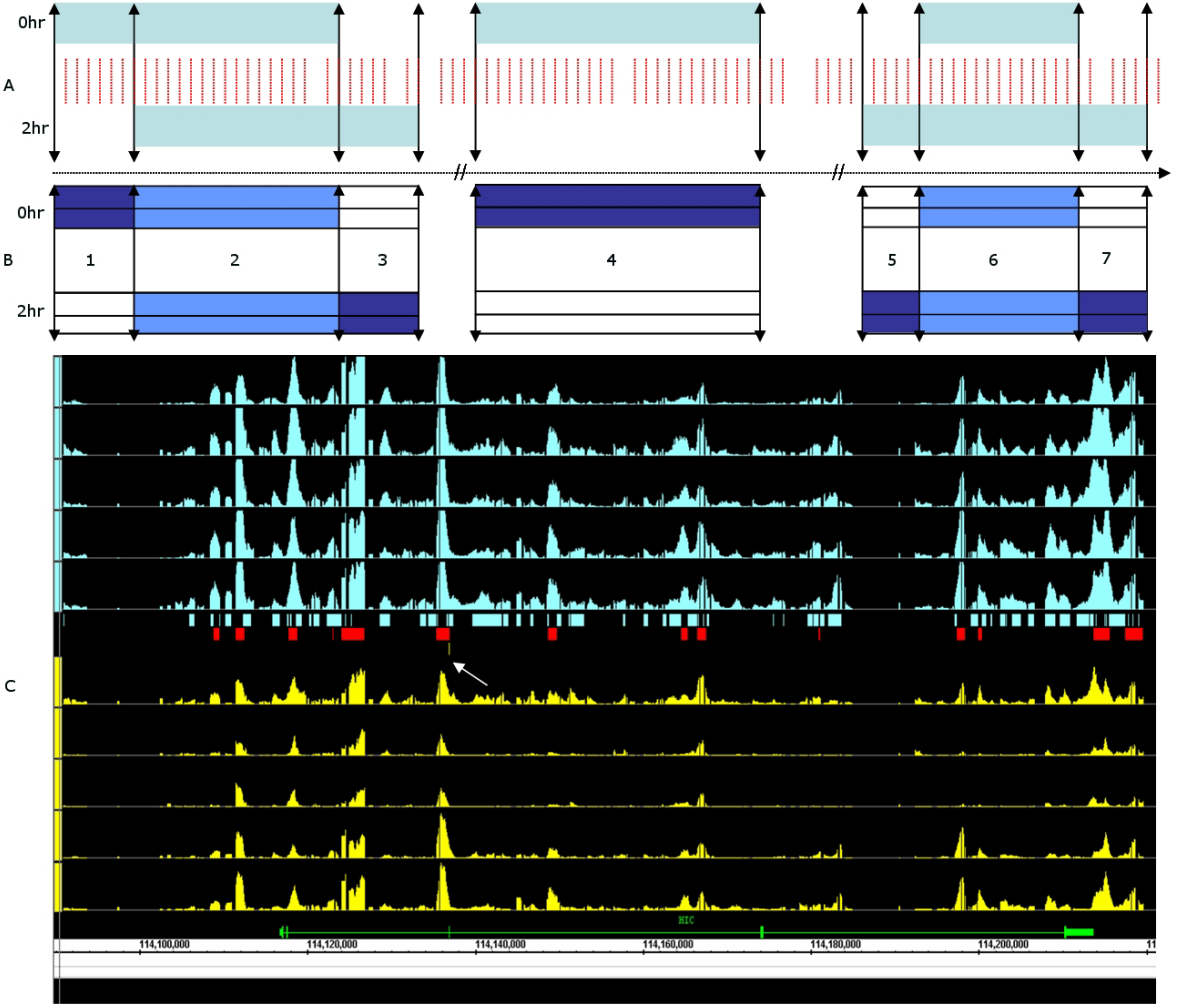
(A-B) A schematic defining FragmentDomainI, FragmentDomainU and NOED. In this example, 0 and 2 hours constitute the reference and target, respectively. In panel A the probes and the enrichment regions are represented in red and light blue respectively; in panel B, the enrichment fragments FragmentDomainU: 1, 3, 5, 7: FragmentDomainI:2, 6; NOED:4. (C): This is an IGB visualization of the fragmented enrichment domains as defined in the reference (blue) and target (yellow). The SE graphs represent biological data from 5 replicates each for reference and target. There are three levels of annotation between the reference and target graphs; the annotations in blue and yellow are representative of enrichment fragments unique to the reference and target, respectively; the annotation in red is representative of the intersecting enrichment fragments. Peaks representative of the binding of putative regulatory elements are evident upstream of and at the 5'end of the HIC gene and in the first few exons and in the introns.

Fig. 1(A–B) presents the schematics of input bounds to gSAM. In this example, 0 and 2 hours constitute the reference and target, respectively. In panel A the probes and enrichment regions are represented by red and light blue bars, respectively; panel B demonstrates the domains defined in Eqn. 15–18; FDU: 1, 3, 5, 7; FDI: 2, 6, NOED: 4. Fig. 1C applies the domain definition to biological data, where the top five SE graphs (blue) are representative of five replicates for the reference and the bottom five graphs (yellow) are representative of the target; all graphs have been scaled identically. Additionally, there are three levels of annotation between the reference and target graphs; the annotations in blue and yellow are representative of enrichment fragments unique to the reference and target, respectively; the annotation in red is representative of the intersecting enrichment fragments. Peaks representative of the binding of putative TREs are evident upstream of and at the 5'end of the *HIC *gene as well as in the first few exons and in the introns. This data is visualized in the Integrated Genome Browser (*IGB*) [52].

The sample-size can be improved by considering probe-specific as opposed to domain-specific input values. This comes at the cost of computational time and potential increase in noise; it requires a post-differential analysis data clustering, followed by the application of a more conservative FDR-based significance threshold for downstream significance analysis. Alternatively, gaussian smoothing of the probe-level data can also enhance the SNR. Finally, it has been validated that the differential transcription/regulation outcome under the probe and domain-specific inputs are consistent with one another (R^2^~0.91). All data presented here, unless specifically noted is generated using domain-specific input. For either types of input, no probe-specific correction is required, since in a pair-wise analysis, signal from identical probes are summarized for both the reference and target samples.

#### V. Summarization of differential response

This section summarizes the differential response of a pair-wise system. This is encapsulated by the four elements in Eqn. 21:

Γ = (**d **- *statistic*,*δ*,*FDR*,*FC*)

**Figure 2:**
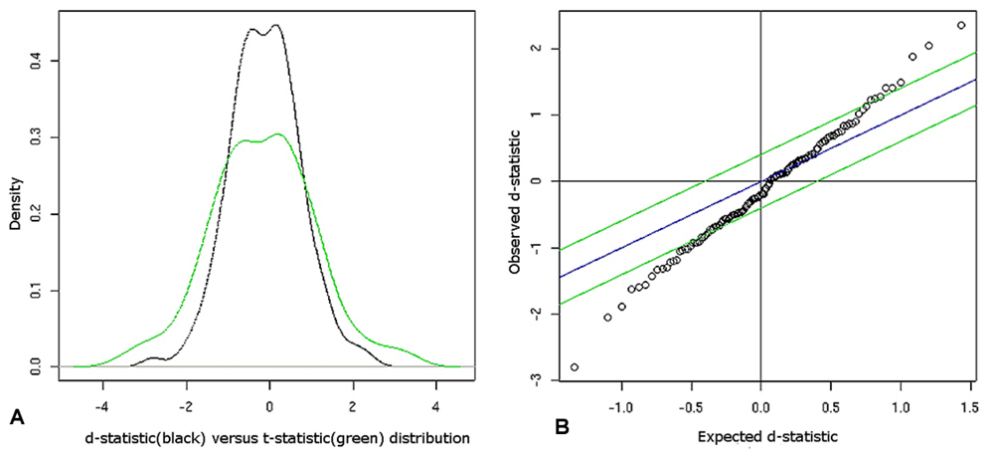
(A) This represents the t-statistic (green) versus d-statistic (black) distribution; the shrinkage in the tails of the latter is due to the additional variance term. (B): This is a scatter-plot of the observed (y-axis) versus expected (x-axis) d-statistic distributions, where the open circles represent the data points. The delta (±Δ) envelope (green) defined about a d-statistic of zero, indicates a null domain – such that regions above and below the positive and negative Δ cutoff indicate up-regulation and down-regulation, respectively.

i) D-statistic: A variant on the t-statistic, it is a standardized differential change index. Fig. 2A presents a comparison of the t-statistic (green) and d-statistic (black) distributions. The additional variance term in the latter is responsible for the shrinkage in the tails, consequently boosting the peak centered about zero. Use of the t-statistic as an estimator of differential change potentially increases the FPR; the d-statistic essentially controls it, thereby optimizing the sensitivity and specificity for differential detection. In the original SAM publication [5], the core bootstrapping step to generate the null d-statistic distribution is carried out across untreated controls and samples treated with ionizing radiation. For ChIP on chip experiments, the labels on the treatment and control replicates are shuffled across the time-pairs in a balanced manner -with equal number of replicates and entries in the reference and target time-pairs – to generate a null (expected) distribution. For RNA transcription the signal intensity across the time-points are permuted to generate the null. Fig. 2B presents the observed (y-axis) versus expected (x-axis) d-statistic distribution.

ii) *δ*: The direction of differential change, often referred to as up or down regulation in the gene expression terminology, is also referred to as positive/negative/null differential shift in gSAM.

iii) FDR: Significance of the differential change is quantified via the FDR or q-value [18,19]. Analogous to the p-value, a measure of significance in terms of the FPR, the q-value is a measure of significance in terms of the FDR. Q-value is the minimal FDR at which a differential change is deemed significant.

iv) FC: Microarray-based FC is a commonly used discriminator for differential change. This essentially estimates true biological change over background by comparing signal intensity/enrichment between the reference and target pair.

Theoretically, any/combination of the output metrics (Γ) can be used for the segmentation of significant versus non-significant differential response. An early method suggested by Tusher *et al *[5] is that of using a delta (±Δ) envelope about a d-statistic of zero, to define a null domain – such that regions above and below the positive and negative Δ cutoff indicate up-regulation and down-regulation, respectively. This is elucidated in Fig. 2B, where the Δ envelope is shown in green. This is a symmetric approach about the d-statistic but does not guarantee symmetric FDR bounds for both up and down regulated regions. Researchers [53] have discussed the dependence of the outcome of SAM, specifically, the variation in the list of significant genes, as a function of the initial threshold. The *Results *discusses the inter-relationship amongst the output metrics, and contrasts the biases introduced by each in the context of transcriptome data.

## Results

The results for the following are presented here: RNA transcription, binding of RNA PolII, and modification of histone factors: HisH4, H3K9K14D (both acetylated), H3K27T (methylated). All samples are hybridized to Affymetrix [20-22,37] ENCODE tiling arrays of 22 bp (average) probe resolution and 10*μ *feature resolution. The ENCODE array interrogates approximately 1 percent of the human genome – a coverage of 15 Mb of the non-repeat portions of the 30 Mb – and does not include regions from chromosomes 3, 17 and Y. Prior to the differential analysis, enriched elements: transfrags [23-25] and putative TREs [43] are identified. The predictive algorithm, gSAM is applied to enriched elements of inter-genic, intronic and exonic origin, encompassing the entirety of protein-coding and non-coding regions. The output of gSAM is a ranked list of differentially changing transfrags or TREs per pair-wise time-point.

The following summarizes the results after application of gSAM:

I. Segmentation metrics for estimation of differential response;

II. Differential expression of annotated and un-annotated transcribed RNA;

III. Differential regulation of putative TREs;

IV. Mono-phasic versus multi-phasic differential regulation clusters;

V. Loci specific examples;

### I. Segmentation metrics for estimation of differential response

This section elucidates the relationship of the segmentation metrics – FC, FDR/q-value and d-statistics.

**Figure 3:**
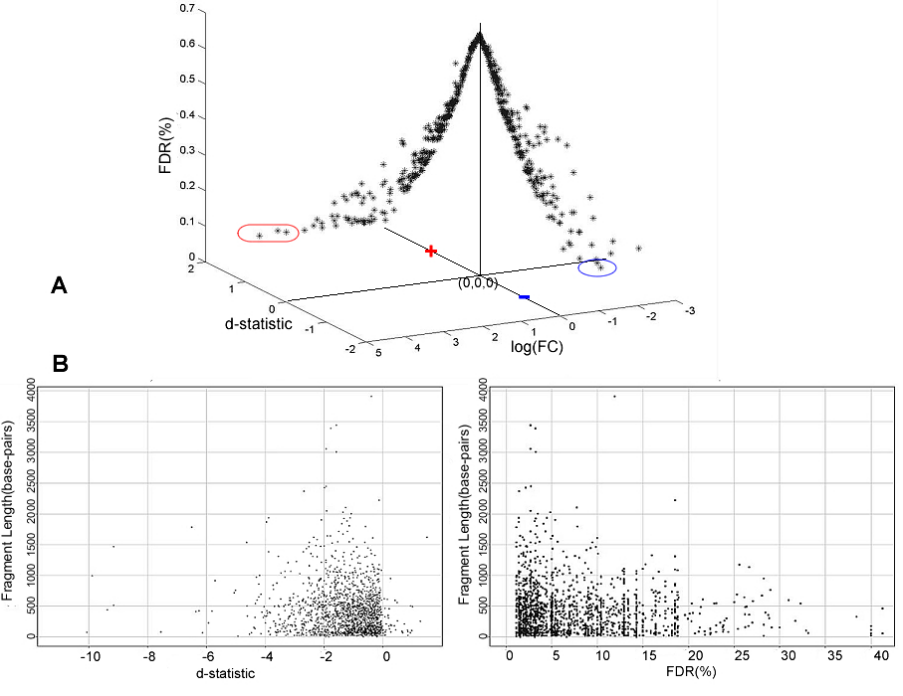
(A) Distribution of the FDR versus d-statistic versus log fold change as shown in the representative 0–2 hour HisH4 data. (B) 0–2 hour HisH4 data, corroborates that no length based bias is introduced the estimation of the d-statistic and/or FDR.

Fig. 3A representing HisH4 differential data at the 0–2 hour interval, but generalizable to all samples, shows the relationship of FDR, d-statistic and logarithmic fold change distributions along the three axes. The data corroborates the following:

i) There is a strong positive correlation between the computed d-statistic and FC;

ii) The FDR(q-value) has an inverse relationship with the absolute value of the d-statistic;

iii) The relationship between FDR and FC is nuanced. Loci (blue oval), exhibiting down regulation (-) at 0.1 percent FDR correspond to a minimum FC of 1.15; in contrast, loci (red oval) exhibiting up-regulation(+) at 0.1 percent FDR correspond to a minimum FC of 4.7. This highlights instances where the arbitrary choice of the 2x FC threshold can result in false positives as well as false negatives.

In general terms, the results underscore the importance of the choice of segmentation metrics, since this affects the gene-significance ranking.

Microarrays tend to compress the real FC; hence an observed small change might indicate a more significant underlying differential. The following results corroborate the stringency of a 2x FC threshold in these transcriptome experiments. For RNA mapping data, the median FC as computed exclusively in exons, across all time-intervals, is 1.59. The median, computed across all transfrags of genic and inter-genic origin is reduced to 1.21–1.35 (across time-intervals). In contrast to the median value (50^th ^percentile), a FC of 2x corresponds to 82^nd ^– 96^th ^percentile (across time intervals); this indicates the introduction of a potentially significant false negative bias, if 2x is used to estimate significant differential change. Similar observations have been made for the differential modification data. For H3K9K14D the median FC ranges from 1.19–1.27 over the time intervals; the 99^th ^percentile values range from 1.62–1.79. The other acetylated histone, HisH4, exhibits slightly higher median FC ranging from 1.22–1.43 with the 99^th ^percentile of the distribution ranging from 1.89–2.14. For RNA PolII, the median range is from 1.28–1.32 with the 99^th ^percentile of the distribution ranging from 1.9–2.36.

Results show a R^2^~0.997 between t-statistic and d-statistic. P-value is however not considered for segmentation of differential change. The existences of multiple cutoffs associated with p-values, which as Lee *et al *[54] describe introduce an artificial binarization of bound-unbound states for each protein interaction. Change in the p-value threshold from 0.001 to 0.05 results in an increase of the regulator-promoter interactions by an order of magnitude. However, the q-value (FDR), which makes use of the bounds on the d-statistic that may be asymmetric (Fig. 3A), is a measure of significance that can be associated with each region.

Finally, it is important to investigate the impact of fragmentation (introduced via domain creation) on the segmentation metrics. Fig. 3B represents 0–2 hour HisH4 data, where the y-axes in the left and right figures correspond to the fragment length and the x-axes correspond to the d-statistic and percent FDR respectively. This corroborates that no length-based bias is introduced in the estimation of the d-statistic and/or FDR (q-value). A predominantly negative shift in the d-statistic bias indicates that there is increased de-acetylation in the 2 hour (target) compared to the 0 hour (reference). Hence the d-statistic is not expected to be symmetric about the point of no change or zero. All differential expression/regulation data discussed from this point forward utilize FDR as the segmentation metric.

### II. Differential expression of annotated and un-annotated transcribed regions

This section summarizes the RNA transcription data.

In these experiments each of the four time points are represented by three biological replicates (B_1_-B_3_), with each sample hybridized in duplicate. Thus gSAM utilizes six samples per time-point. The median R^2 ^across all replicates and over all time intervals is 0.9 with a median slope of 1.12, attesting to high reproducibility across samples.

The analyses identify and quantify distinctly different temporal and spatial expression profiles. The highest and lowest fraction of differential expression, when summarized across all transfrags, is observed during the 8–32 hour and 2–8 hour time intervals, respectively. Down-regulation dominates the former and up-regulation the latter interval. Complete results are tabulated in Table 2. On the whole, down-regulation is statistically more significant and 2.4–2.8 percent of the down-regulated fraction has a FDR less than 12 percent. The annotated transfrags demonstrate dominant up-regulation throughout the entire time-course, with 16x (16.17 versus 0.52) higher up-regulation, at FDR less than 12 percent, observed between 0–2 hours. Transfrags in the non-coding regions and introns demonstrate a dominant down-regulation at comparable statistical significance. The piece-wise model of gSAM highlights the observation that not all exons of a transcript demonstrate consistent FC. The observed variance in differential expression across exons of transcripts is reduced from 2.25 to 0.25 upon exclusion of terminal exons. Since the assay is not strand specific, it can be hypothesized that the increased variance may reflect effects of differential expression arising from overlapping transcripts. The hypothesis has to be validated via experiments such as strand-specific Northern blots.

**Table 2:** Temporal differential expression profile observed in RNA transcription study

**Time-Interval**	**Source**	**Observed Differential Expression**	**Fraction: Down Regulated**
0–2 hour	All transfrags (genic+intergenic)	34%	62% of 34%
2–8 hour	All transfrags	19.75%	28% of 19.75%
8–32 hour	All transfrags	53.8%	53% of 53.8%

**Figure 4:**
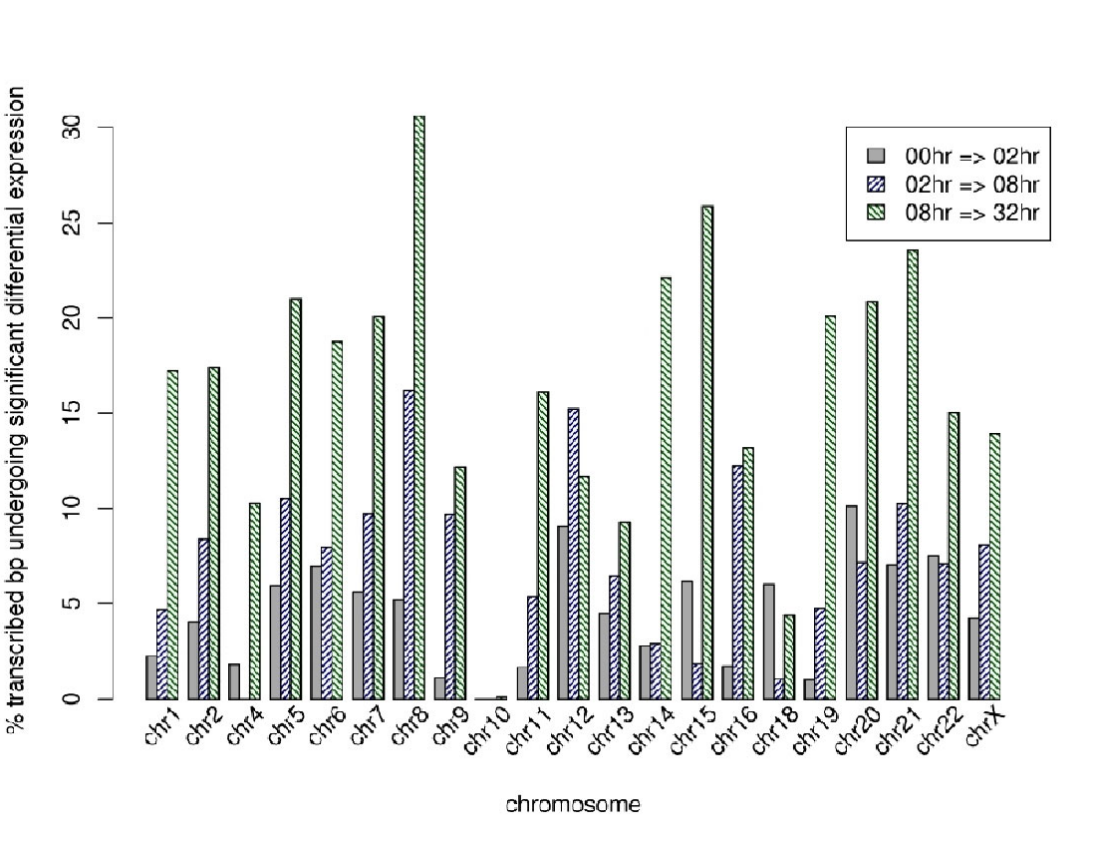
The histogram summarizes the differential expression profiles in each ENCODE region on each chromosome. Chromosome region specific differential expression is observed across the time-points – 30 percent change on chromosome 8 to no detectable change on chromosome 10. Globally, the highest fraction of differential expression when summarized across all transfrag is observed between 8–32 hours (53.8 percent),. The most statistically significant (FDR ≤12 percent) changes are also observed between 8–32 hours.

The differential responses across the ENCODE regions vary significantly. The changes in the expression level range from approximately 30 percent of the interrogated bases on chromosome 8 to un-detectable in that on chromosome 10. The general trend that is consistent across the chromosomes is an increase in the percent bp that is differentially expressed as a progression of time, as summarized in Fig. 4. This is potentially due to the fact that as the time intervals increase, the observed differential response incorporates residual changes from the prior state(s) – upholding the assumption of an accumulator system in gSAM.

### III. Differential regulation of putative TREs

This section summarizes the ChIP-chip data.

For modified histones and RNA PolII each of the four time points are represented by five biological replicates (B_1_-B_5_) with each sample hybridized in duplicate. Thus gSAM utilizes ten samples per time-point. The reproducibility in the ChIP on chip samples is more variable compared to the RNA samples. For HisH4 across all time-points, R^2 ^ranges from 0.6–0.71. For H3K27T, R^2 ^ranges from 0.54–0.7 with 32 hour contributing to the low end. For H3K9K14D, R^2 ^ranges from 0.6–0.77 with maximal variation at 32 hours. For RNA PolII, R^2 ^is approximately 0.53 percent. In all cases, the ENCODE regions on chromosome 4, which only interrogates un-annotated regions, is a significant contributor to the low end of the correlation distribution. While the permutative framework in gSAM provides resilience against the variance, the overall reduced reproducibility does affect the outcome by resulting in an increased FDR. This can introduce a false negative bias in the segmentation of differential sites. This bias can be exacerbated, if poor reproducibility is coupled with too few replicates available for permutation. This premise has been tested in a simulation experiment where inter-replicate reproducibility is reduced via artificial introduction of noise such that the R^2 ^for HisH4 is reduced to <0.50. This resulted in an average increase of FDR by 6 percent.

**Figure 5:**
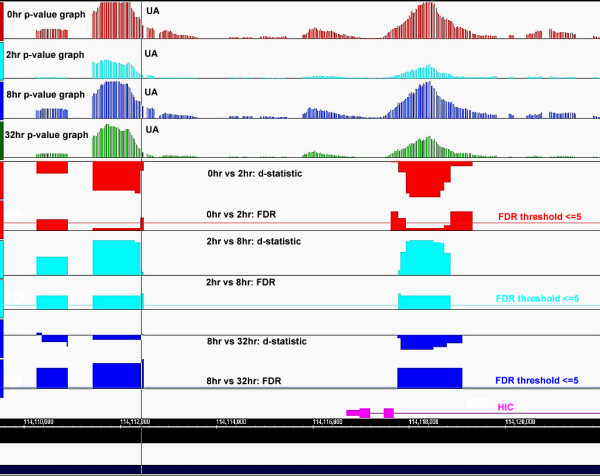
D-statistic versus FDR relationship at putative TREs, across the time-series (IGB view). Examples of enrichment fragments are observed within and upstream of the second intron of the HIC gene (pink). The upstream fragment is possibly un-annotated (UA), in so far as no RefSeq annotation is available. The top four tracks represent the HisH4 p-value graphs at 0 (red), 2 (light-blue), 8 (dark-blue) and 32 (green) hours, scaled appropriately for comparison; the subsequent tracks represent the d-statistic (top) and FDR (bottom) pair for the 0–2 (red), 2–8 (cyan) and 8–32 (blue) hour time intervals. The horizontal lines associated with the FDR data refer to the 5 percent threshold in each case.

The IGB visualization in Fig. 5 shows an example of enrichment fragments within and upstream of the second intron of the *HIC *gene (pink). The upstream fragment is possibly un-annotated (UA), in so far as no RefSeq annotation is available. The top four tracks represent the HisH4 p-value graphs at 0 (red), 2 (light-blue), 8 (dark-blue) and 32 (green) hours, scaled appropriately for comparison; the subsequent track-pairs represent the d-statistic (top) and FDR (bottom) for the 0–2 (red), 2–8 (cyan) and 8–32 (blue) hour time intervals. The horizontal lines associated with the FDR data demarcate the 5 percent threshold.

There are four salient observations, in this data:

i. The putative TREs at the 5'end and upstream of the 5'end of *HIC *exhibit temporally distinct differential regulation profiles. For the 0–2 hour interval both manifest down-regulation, followed by up-regulation between 2–8 hours and subsequent down-regulation between 8–32 hours. This differentiation would not have been evident if broader time-intervals were selected, attesting to the importance of the temporal resolution in overall experimental design.

ii. The piece-wise model in gSAM facilitates tracking of the variable levels of differential regulation throughout a putative TRE, as well as the associated modulation in FDR. The 0–2 hour interval the most significant (less than 5 percent FDR) differential change is associated with the peak of the d-statistic in the second intron. This is not afforded by SAM in the current mode.

iii. Although no annotation is available for the differential regulation observed upstream of *HIC*, the observed differential activity is also significant at less than 5 percent FDR (0–2 hours). Due to the underlying permutative framework the FDR estimates of the novel and known regions are on par with one another. This putative and novel TRE constitutes a perfect co-regulation candidate for validation via alternative biochemical means.

iv. gSAM is a signal enrichment based metric, but as is evident from the figure, there is a strong correlation – R^2 ^> 0.965 – with the p-value based enrichment peaks [42].

While a single example is presented above, the observations can be generalized across the genome. In general, the d-statistic defines the footprint of the putative TRE, and the FDR differential, frequently facilitates identification of the peak of the TRE.

Very few of the biological factors show significant differential change at 5 percent FDR. The following summarizes the significant changes observed in interrogated genic annotation, which is inclusive of exons, introns, UTRs and 250 base-pairs upstream and downstream of 5' and 3' ends respectively. For predictions at 5 percent FDR, among the histone factors HisH4 shows maximal change; 22.9 percent of interrogated genic annotation manifest down-regulation between 0–2 hours, followed by 6 percent exhibiting up-regulation during 2–8 hours. No significant changes are observed between 8–32 hours. For RNA PolII maximal changes are observed between 2–8 hours; at 5 and 7 percent FDR approximately 1 and 5 percent of the interrogated genic annotation exhibit up-regulation, respectively; the observed increase in differentially regulated loci potentially implies that the choice of 5 percent FDR might be too stringent for the case of RNA PolII. On the basis of loci-level coverage, the ranked list of factors undergoing significant differential change is: HisH4 >> RNA PolII > H3K27D, H3K9K14D. A catalog of loci-level gSAM predictions of differential regulation have been presented in Section V.

### IV. Mono-phasic versus multi-phasic differential regulation clusters

This section discusses the classification of the observed differential modification pattern.

The differential pattern observed in the pair-wise analysis can be broadly classified into the following three phases (summarized in Eqn. 22):

i. A positive differential shift(*δ*_+_) is indicative of increased activity in the target with respect to the reference;

ii. A negative shift(*δ*_-_) is indicative of the converse;

iii. A null shift (*δ*_null_), is indicative of no change in enrichment response.

δ+=μtarg⁡et−μreference>0δ−=μtarg⁡et−μreference<0δnull=μreference−μtarg⁡et→0
 MathType@MTEF@5@5@+=feaafiart1ev1aaatCvAUfKttLearuWrP9MDH5MBPbIqV92AaeXatLxBI9gBaebbnrfifHhDYfgasaacH8akY=wiFfYdH8Gipec8Eeeu0xXdbba9frFj0=OqFfea0dXdd9vqai=hGuQ8kuc9pgc9s8qqaq=dirpe0xb9q8qiLsFr0=vr0=vr0dc8meaabaqaciaacaGaaeqabaqabeGadaaakeaafaqaaeWabaaabaacciGae8hTdq2aaSbaaSqaaiabgUcaRaqabaGccqGH9aqpcqWF8oqBdaWgaaWcbaGaemiDaqNagiyyaeMaeiOCaiNaei4zaCMaemyzauMaemiDaqhabeaakiabgkHiTiab=X7aTnaaBaaaleaacqWGYbGCcqWGLbqzcqWGMbGzcqWGLbqzcqWGYbGCcqWGLbqzcqWGUbGBcqWGJbWycqWGLbqzaeqaaOGaeyOpa4JaeGimaadabaGae8hTdq2aaSbaaSqaaiabgkHiTaqabaGccqGH9aqpcqWF8oqBdaWgaaWcbaGaemiDaqNagiyyaeMaeiOCaiNaei4zaCMaemyzauMaemiDaqhabeaakiabgkHiTiab=X7aTnaaBaaaleaacqWGYbGCcqWGLbqzcqWGMbGzcqWGLbqzcqWGYbGCcqWGLbqzcqWGUbGBcqWGJbWycqWGLbqzaeqaaOGaeyipaWJaeGimaadabaGae8hTdq2aaSbaaSqaaiabd6gaUjabdwha1jabdYgaSjabdYgaSbqabaGccqGH9aqpcqWF8oqBdaWgaaWcbaGaemOCaiNaemyzauMaemOzayMaemyzauMaemOCaiNaemyzauMaemOBa4Maem4yamMaemyzaugabeaakiabgkHiTiab=X7aTnaaBaaaleaacqWG0baDcyGGHbqycqGGYbGCcqGGNbWzcqWGLbqzcqWG0baDaeqaaOGaeyOKH4QaeGimaadaaaaa@8F0D@

At any time-interval the putative TREs for most factors show a mixture of the above-defined differential phases. This defines two or more population of loci, where a fraction, *f*_*1*_, up-regulated, *f*_*2*_, down-regulated and (*1*- *f*_*1 *_- *f*_*2*_)remain unchanged. This results in two clusters: mono-phasic or one with a near homogenous differential change, where *f*_*1 *_>> *f*_*2 *_(vice-versa) and multi-phasic which exhibits a mixture of phases. Mathematically, multi-phasic modes are estimated by fitting greater than one gaussian curve to the d-statistic distribution. While this observation is not novel, gSAM provides a tool to identify and quantify these different phases. In reality, no factors exhibit a purely mono-phasic mode; RNA PolII, and the acetylated histones -HisH4 and H3K9K14D – are representative of a distribution where the mono-phasic mode is the dominant one. In contrast, the methylated histone – H3K27T – manifests a distinctly mixed distribution. Furthermore, the loci can switch between the mono-phasic and multi-phasic modes across different time intervals.

**Figure 6:**
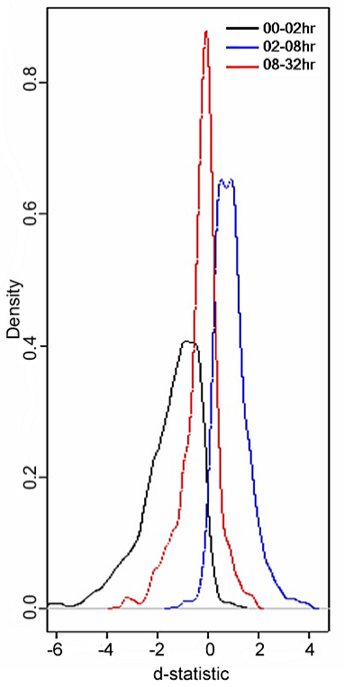
Example: For HisH4 a certain percentage of loci manifest up-regulation, while others manifest down-regulation and yet others exhibit no differential change. The time intervals 0–2 hr, 2–8 hr and 8–32 hr are shown in black, blue and red respectively.

Fig. 6 is representative of the density estimate (based on a gaussian kernel) of the d-statistic distribution for HisH4 which approximates a mono-phasic behavior. It is evident that in the 0–2 hour (black) interval, a significant percentage of the loci manifest, *δ*_- _phase, as indicated by the mode of the d-statistic at -1.7 with a heavy left tail. Between 2 and 8 hours (blue) the mode is shifted to +1.6 with a heavy right tail, indicative of a pre-dominant *δ*_+ _phase or up-regulation at 8 hours. The mode for the 8–32 hour (red) interval approximates the null shift but still exhibits a predominant down regulation. In the biological context of quantifying differential modification of tetra-acetylated histone (HisH4), *δ*_+ _and *δ*_- _are potentially indicative of acetylation and de-acetylation respectively, in response to stimulation by ATRA.

**Figure 7:**
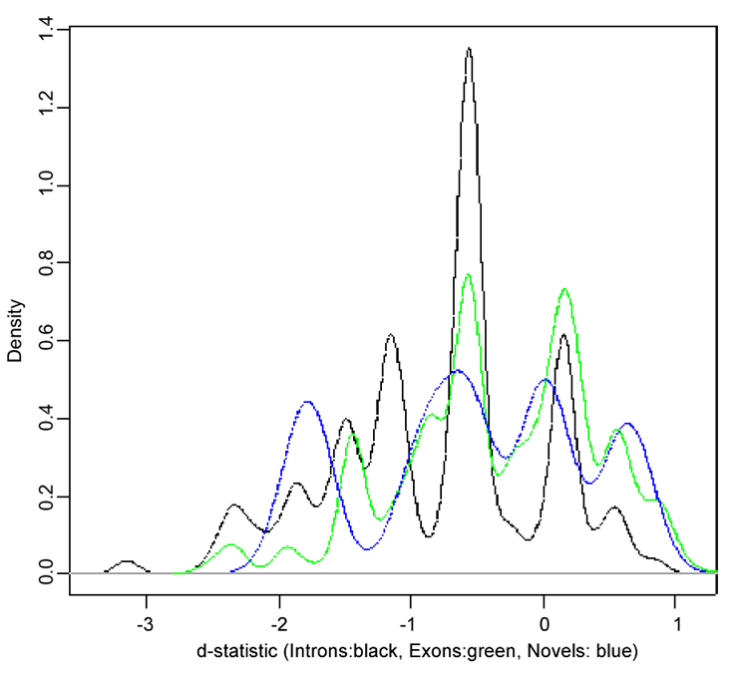
A representative density profile of the d-statistic for change in H3K27T histone modification between 0 and 2 hours of retinoic acid treatment for the ENCODE region on chromosome 1. The curves of different colors illustrate differential change for the H3K27T modification in exonic (green), intronic (black) and intergenic (blue) regions. The shift into the negative territory for the d-statistic for all classes of regions suggest is a consistent downward trend for this modification between 0 and 2 hours.

Fig. 7 is shows the density estimate of the d-statistic distribution for H3K27T in the exons (green), introns (black) and un-annotated (blue) regions of an ENCODE region on chromosome 1. At the chromosomal level of organization, the observed differential modification trends for H3K27T across all annotation types (exon/intron/un-annotated) are consistent; it shows pre-dominant down-regulation as evident by the centering about -0.7. It is conceivable that for other chromosomes/factors there is a dominant phase-discordance, identifying loci where potentially inhibitive and non-inhibitive regulatory mechanisms are at play. gSAM analysis provides a tool to identify and de-convolute the differential effects of these mechanisms.

There is significant co-localization, in the genome, of the differential binding enrichment observed for RNA PolII and HisH4. The significance is highest at p-value <0.003 between 0–2 and 2–8 hours intervals. Over 8–32 hours the p-value while still significant drops to 0.01–0.03. This significance is particularly striking in view of the fact that the array based FC is essentially less than two-fold. There is substantial overlap between the differentially modified regions for RNA PolII and the acetylated histone – HisH4. The overlap can be partitioned into genic (RefSeq-based) and inter-genic regions, encompassing 65 and 35 percent, respectively. The coverage of the genic partition extends across 45 percent of all genes interrogated on the ENCODE array. Using the piece-wise model in gSAM, the genic overlap is further partitioned to estimate the 5' versus 3' versus intra-genic bias and the results have been summarized in Table 3. Bootstrapping establishes the pre-dominant 5' bias of 73 percent is significant at p-value < 10^-13^.

**Table 3:** Overlapping differential modification in genic versus inter-genic regions

	**Overlap: Inter- genic**	**Overlap: Genic**	**Overlap Coverage: % genes profiled by the ENCODE array**	**Overlap: 5'end**	**Overlap: Internal to genes**	**Overlap: 3'end**	**p-value: 5'end overlap**
RNAPolII- HisH4	45%	65%	45%	73.1%	51.9%	7.7%	10^-13^
RNAPolII- H3K27T	48.11%	51.89%	20.1%	57.5%	30%	12.5%	10^-7^

In a global sense, the dominant phase of the differential binding of the methylated factor (H3K27T) is anti-correlated with RNA PolII and the acetylated factor (HisH4). H3K27T and RNA PolII exhibit an overall 51.89 percent co-localization in genic regions, but the coverage of the genic partition encompasses only 20.1 percent of all the genes profiled by the ENCODE array. Bootstrapping establishes the pre-dominant 5' bias of 57.5 percent is significant at a p-value < 10^-7^; results are summarized in Table 3. There is less than 1 percent co-localization between HisH4 and H3K27T; this makes biological sense since the former is associated with active genes while the latter is associated with repressed, silenced genes.

### V. Loci-specific examples

This section outlines certain clustering strategies for differentially changing loci.

The gSAM model together with the FDR based segmentation provides a powerful tool to generate a predictive list and clustering of loci that undergo differential modification. Some examples are:

i) Intra or inter-factor segmentation within a specified/across different time-interval(s);

ii) Intra and inter-factor segmentation FDR and/or d-statistics based;

iii) Segmentation based on inter-factor co-regulation pattern.

Table 4 lists all the overlapping loci (on opposite strands) that show significant differential binding for HisH4 at different time intervals. These loci are potentially reflective of bi-directional regulation activity, although further experimental validation is required. The caveat to this analysis is that this predictive list includes only loci where greater than 90 percent of the probes or enrichment domains exhibit consistent co-regulation patterns – potentially introducing a false negative bias. The FDR threshold for the analysis is set at 5 percent, although there are a few instances where loci representing FDR as high as 6.7 percent have also been included; this tolerance is allowed for cases where at this cutoff a sharp increase in FDR to greater than 15 percent is observed. The instances listed as *NA *are representative of cases where the observed differential modification is at a significantly higher FDR as compared to the counterpart loci on the opposite strand. The *Annotation *segregates the data obtained from domains that are common to the overlapping loci ("+"), versus the domains that are unique to each locus. For the majority, the differential changes observed in the unique and intersecting domains are consistent in strength (*diff change*) and direction of change (*Acetylation summary*). Also, for the majority, opposing differential trends are observed between the 0–2 hours and 2–8 hours time intervals. Functional definition assembled from Gene Ontology data can be used to augment this clustering.

**Table 4:** HisH4 – Ranked list of differentially changing loci on overlapping strands, generated with a 5%FDR threshold

**Factor**	**Time Interval**	**Chr**	**Annotation**	**Strand**	**Diff change**	**Acetylation Summary**	**%FDR**	**GO**
HisH4	00_02	1	TCFL1	AS	-1.539	-	3.43	Transcription Factor Like1
HisH4	00_02	1	TCFL1 + PIP5K1A		-2.166	-	2.70	
HisH4	00_02	1	PIP5K1A	S	-2.373	-	2.72	Signal transduction
HisH4	02_08	1	TCFL1	AS	1.343	+	6.74	
HisH4	02_08	1	TCFL1 + PIP5K1A		1.545	+	6.74	
HisH4	02_08	1	PIP5K1A	S	NA	NA	NA	

HisH4	00_02	5	GDF9	AS	NA	NA	NA	Growth differentiation factor9
HisH4	00_02	5	GDF9 + QP-C		-1.129	-	4.11	
HisH4	00_02	5	QP-C	S	NA	NA	NA	Part of the mitochondrial respiratory chain

HisH4	00_02	5	FGF1	AS	-1.169	-	2.67	Fibroblast growth factor
HisH4	00_02	5	FGF1 + ARHGAP26		-1.169	-	2.67	
HisH4	00_02	5	ARHGAP26	S	-1.502	-	2.67	Rho GTPase activating protein

HisH4	00_02	6	C6orf150 D24	AS	NA	NA	NA	
HisH4	00_02	6	C6orf150+MTO1		-2.857	-	2.31	
HisH4	00_02	6	MTO1	S	-2.623	-	2.31	
HisH4	02_08	6	C6orf150	AS	NA	NA	NA	MTO1: Cellular respiration
HisH4	02_08	6	C6orf150+MTO1		1.786	+	4.04	
HisH4	02_08	6	MTO1	S	1.695	+	4.04	

HisH4	00_02	6	SNX3	AS	-2.600	-	2.31	Intracellular signalling cascade
HisH4	00_02	6	LACE1	S	-1.954	-	4.35	Unknown
HisH4	02_08	6	SNX3	AS	-0.849	-	4.04	
HisH4	02_08	6	SNX3+LACE1		0.828	+	4.04	
HisH4	02_08	6	LACE1	S	0.849	+	4.04	

HisH4	00_02	7	GCC1	AS	NA	NA	NA	DNA binding protein
HisH4	00_02	7	GCC1 + ARF5		-1.574	-	1.54	
HisH4	00_02	7	ARF5	AS	NA	NA	NA	Enzyme activator activity

HisH4	00_02	22	EIF4ENIF1	AS	NA	NA	NA	Translation initiation factor
HisH4	00_02	22	EIF4ENIF1 + SFI1		-1.407	-	4.95	
HisH4	00_02	22	SFI1	S	NA	NA	NA	Spindle assembly associated(yeast)

HisH4	00_02	22	SYN3	AS	NA	NA	NA	Cell-cell signaling
HisH4	00_02	22	SYN3+TIMP3		-1.253	-	4.95	
HisH4	00_02	22	TIMP3	S	NA	NA	NA	Enzyme Inhibitor activity

HisH4	00_02	21	GART	AS	-2.343	-	2.18	Cellular biosynthesis/catalytic activity
HisH4	00_02	21	GART+SON		-2.120	-	2.28	
HisH4	00_02	21	SON	S	-1.000	-	5.03	DNA binding

HisH4	00_02	X	CXorf12	AS	-0.855	-	3.18	
HisH4	00_02	X	CXorf12+IRAK1		-0.840	-	3.09	
HisH4	00_02	X	IRAK1	S	-1.234	-	1.87	Interleukin-1 receptor binding

Tables 5, 6 provide a loci-specific ranking based on FDR threshold. These are potentially optimal candidates for biochemical validation. In the analysis presented here only the top 10^th ^percentile at a 5 percent FDR is shown. It is an interesting observation that the rank ordering of the loci is not preserved across time-points. This could potentially reflect variable functions of loci at different states of development. This methodology provides a tool for the study of the developmental transcriptome, for instance.

**Table 5:** HisH4: Ranked list of differentially changing loci between 0–2 hours. The list is generated using a 5% FDR

**Factor**	**TimePoints(hours)**	**Chromosome**	**Ranked Annotation**
HisH4	00_02	21	ATP50
HisH4	00_02	21	C21orf49
HisH4	00_02	21	C21orf55
HisH4	00_02	21	CRYZL1
HisH4	00_02	21	IFNAR2
HisH4	00_02	21	WDR9
HisH4	00_02	21	WRB
HisH4	00_02	21	SH3BGr
HisH4	00_02	21	C21orf4
HisH4	00_02	21	C21orf119
HisH4	00_02	21	HMGN1
HisH4	00_02	21	C21orf62
HisH4	00_02	7	CTNBP2
HisH4	00_02	7	GRM8
HisH4	00_02	7	HIC
HisH4	00_02	7	LOC85865
HisH4	00_02	7	ST7
HisH4	00_02	21	IL1ORB
HisH4	00_02	X	BIRC4
HisH4	00_02	X	MECP2
HisH4	00_02	21	C21orf59
HisH4	00_02	21	ITSN1
HisH4	00_02	21	IFNAR1
HisH4	00_02	21	FLJ46020

**Table 6:** HisH4: Ranked list of differentially changing loci between 2–8 hours. The list is generated using a 5% FDR

**Factor**	**TimePoints(hours)**	**Chromosome**	**Ranked Annotation**
HisH4	02_08	6	C6orf148
HisH4	02_08	6	C6orf150
HisH4	02_08	6	C6orf49
HisH4	02_08	6	DDX43
HisH4	02_08	6	EEF1A1
HisH4	02_08	6	FOXP4
HisH4	02_08	6	FRS3
HisH4	02_08	6	LACE1
HisH4	02_08	6	MGC20741
HisH4	02_08	6	MT01
HisH4	02_08	6	OSTM1
HisH4	02_08	6	SEC63
HisH4	02_08	6	SNX3
HisH4	02_08	6	TFEB
HisH4	02_08	6	KCNQ5
HisH4	02_08	12	SLC2A13
HisH4	02_08	1	KIAA1441
HisH4	02_08	1	PIK4CB
HisH4	02_08	1	POGZ
HisH4	02_08	1	PSMB4
HisH4	02_08	1	PSMD4
HisH4	02_08	1	RFX5
HisH4	02_08	1	SNX27
HisH4	02_08	1	TCFL1

Some examples of clustering based on inter-factor co-regulation are listed below. The most significant (listed in order) differential changes in both HisH4 and RNA PolII are observed at the following genes: *PIK4CB *(chr. 1) > *DDX18 *(chr. 2) > *STK11IP *(chr. 2) >*SNX27 *(chr. 2). *PCDH15 *(chr. 10) exhibits a significant differential binding site exclusively between the 2–8 hour intervals. RNA PolII and H3K27T exhibit co-binding to *TRPM8 *(chr. 2). Non-overlapping binding sites are observed for HisH4 and H3K27T on the *GRM8 *(chr. 7). These observations are significant at an FDR ≤12 percent.

## Discussion

gSAM can be applied to any type of differential mechanism experiment; the differential changes can be predicted, partitioned and ranked at any level – genic, sub-genic and/or inter-genic. An exclusively gene-level estimate, as with SAM, does not have the sensitivity to determine these changes. While a FDR of 5 percent has been used for segmentation, under certain scenarios this might be too stringent. If a known TRE is not predicted by gSAM it simply implies that element does not manifest a differential change under the FDR threshold used for data segmentation. An optimal threshold estimate is to determine the steepest gradient in the FDR distribution and consider its mid-point as the ideal value.

There is a caveat to the piece-wise model; it does not track the change of individual probe membership in genic components according to the expression of spliced isoforms. It does not track and hence account for the possibility that an individual probe can and probably is measuring overlapping and yet different transcripts. Analogous to all differential analysis, gSAM is based on the principle of co-regulation of a probe-set. If different overlapping transcripts have variable direction of change, either the effects cancel out or the resultant change represents the predominant trend similar to a *majority rule *in a complex background. The majority rule also governs the behavior of a probe-set with mixed membership. The overall outcome depends on the concordance of change of different transcripts represented by different probes in the piece-wise assignment and the abundances of the changing transcripts. It will likely be different in different scenarios.

The purpose of the manuscript is to detail algorithms for predictions of differentially changing loci. The bootstrapping outcome discussed in *Results *provides computational validation of gSAM. Quantitative PCR (qPCR) is a biochemical alternative that can be used for validation. The comparison between qPCR validation and the array-based gSAM predictions is qualitative in most regards; in making conclusions, due consideration should be given to the nuances discussed. qPCR discriminates at 95 percent sensitivity [43] between an enrichment site (differentially changing or not) and a non-site, and potentially validates the direction of the differential change. However, there is no mechanism to precisely equate the fold changes as measured by qPCR and by microarrays. The qPCR and array-based metrics do not follow a linear relationship; the correlation between the two improves at highly significant array-based p-values < 10^-7 ^[43]. This discordance between the two is partially because array hybridizations are performed on amplified DNA, while qPCR is frequently performed on non-amplified immunoprecipitated DNA. Consequently, qPCR fold change cannot be directly equated to the gSAM-based d-statistics. Finally, the output from gSAM is a relative measure of signal accumulated in response to an external stimulus, wherein the change is profiled at two time points. Therefore, it is important that the qPCR validation be performed at exactly the same time-points for the same replicates – otherwise data interpretation might be difficult to impossible.

## Conclusion

gSAM provides a powerful extension to SAM by facilitating the exploration of differential regulation in an unbiased and annotation independent manner. The assumption of an underlying piece-wise model enables the isolation of regions of maximal or peak differential change. These regions can be observed in protein-coding as well as non-coding regions. Since the proposed method does not have a coding bias and uses a FDR-based metric for segmentation of differential regulation, it provides a predictive mechanism to generate a ranked list of regions that can be validated by alternative biochemical means such as qPCR. The FDR-based segmentation also facilitates comparison of differentially changing loci across different microarray platforms. The above gSAM predictions provide some evidence for dynamic changes in the transcriptional regulatory elements. The changes are maximal in the acetylated histone H4. The correlation of the temporal trends in the other factors with HisH4 indicates the occurrence of similar dynamics, the exact behavior of which will need to be validated. Nonetheless, the FDR-ranked differentially changing loci provide a short-list of predictions of dynamically changing transfrags and TREs in the ENCODE region.

## Authors' contributions

SG developed and implemented the algorithm; all code is written in R [55] version 2.0.1 [see Additional file 1]. HAH and EAS were involved in ChIP sample generation. PK was involved in RNA mapping experiments. TRG and KS were involved in sample and array data generation, discussion of data analysis and overall guidance in the project. SG wrote the manuscript and all authors read and approved the final version.

## Supplementary Material

Additional file 1 gsam_prototypercode.zip. File archive comprising of prototype R code for gSAM implementation including readme and examples. Click here for file

## References

[B1] Elvidge G (2006). Microarray expression technology: from start to finish. Pharmacogenomics.

[B2] Zhang X, Kluger Y, Nakayama Y, Poddar R, Whitney C, Detora A, Weissman SM, Newburger PE (2004). Gene expression in mature neutrophils: early responses to inflammatory stimuli. Journal of leukocyte biology. Journal Leukoc Biol.

[B3] Werner SL, Barken D, Hoffmann A (2005). Stimulus Specificity of Gene Expression Programs Determined by Temporal Control of IKK Activity. Science.

[B4] Grigoryev DN, Ma SF, Irizarry RA, Ye SQ, Quackenbush J, Garcia JGN (2004). Orthologous gene-expression profiling in multi-species models search for candidate genes. Genome Biology.

[B5] Tusher VG, Tibshirani R, Chu G (2001). Significance analysis of microarrays applied to the ionizing radiation response. PNAS.

[B6] Strimmer K (2003). Modeling gene expression measurement error: a quasi-likelihood approach. BMC BioInformatics.

[B7] Benjamini Y, Hochberg Y (1995). Controlling the False Discovery Rate: a Practical and Powerful Approach to Multiple Testing. Journal of the Royal Statistical Society B.

[B8] Dudoit S, van der Laan MJ, Pollard KS (2004). Multiple Testing. Part I. Single-Step Procedures for Control of General Type I Error Rates. Statistical Applications in Genetics and Molecular Biology.

[B9] van der Laan MJ, Dudoit S, Pollard KS (2004). Multiple testing. Part II. Step-down procedures for control of the family-wise error rate. Statistical Applications in Genetics and Molecular Biology.

[B10] Pounds SB (2006). Estimation and control of multiple testing error rates for microarray studies. Briefings in Bioinformatics.

[B11] Dudoit S, Yang YH, Callow MJ, Speed TP (2000). Statistical Methods for Identifying Differentially Expressed Genes in Replicated cDNA Microarray Experiments. Technical Report # 578.

[B12] Benjamini Y, Hochberg Y (2000). On the Adaptive Control of the False Discovery Rate in Multiple Testing with Independent Statistics. Journal of Educational and Behavioral Statistics.

[B13] Holm S (1979). A Simple Sequentially Rejective Bonferroni Test Procedure. Scandinavian Journal of Statistics.

[B14] Westfall PH, Young SS (1993). Resampling-based Multiple Testing.

[B15] Benjamini Y, Yekutieli D (2001). The Control of the False Discovery Rate in Multiple Testing under Dependency. The Annals of Statistics.

[B16] Yekutieli D, Benjamini Y (1999). Resampling-based False Discovery Rate Controlling Multiple Test Procedures for Correlated Test Statistics. Journal of Statistical Planning and Inference.

[B17] Efron B, Tibshirani R, Storey JD, Tusher V (2001). Empirical Bayes Analysis of a Microarray Experiment. Journal of the American Statistical Association.

[B18] Storey JD (2002). A Direct Approach to False Discovery Rates. Journal of the Royal Statistical Society, Series B.

[B19] Storey JD, Taylor JE, Siegmund D (2004). Strong Control, Conservative Point Estimation, and Simultaneous Conservative Consistency of False Discovery Rates: A Unified Approach. Journal of the Royal Statistical Society, Series B.

[B20] The ENCODE Project Consortium (2004). The ENCODE Project. Science.

[B21] The ENCODE datasets can be downloaded from the following website. http://genome.ucsc.edu/ENCODE/encode.hg17.html.

[B22] The ENCODE Project Consortium (2007). The ENCODE pilot project: identification and analysis of functional elements in 1 percent of the human genome. Nature.

[B23] Kapranov P, Cawley SE, Drenkow J, Bekiranov S, Strausberg RL, Fodor SPA, Gingeras TR (2002). Large Scale Transcriptional Activity in Chromosomes 21 and 22. Science.

[B24] Cheng J, Kapranov P, Drenkow J, Dike S, Brubaker S, Patel S, Long J, Stern D, Tammana H, Helt G, Sementchenko V, Piccolboni A, Bekiranov S, Bailey DK, Ganesh M, Ghosh S, Bell I, Gerhard DS, Gingeras TR (2005). Transcriptional Maps of 10 Human Chromosomes at 5-Nucleotide Resolution. Science.

[B25] Kampa D, Cheng J, Kapranov P, Yamanaka M, Brubaker S, Cawley SE, Drenkow J, Piccolboni A, Bekiranov S, Helt G, Tammana H, Gingeras TR (2004). Novel RNAs Identified from an In-depth Analysis of the Transcriptome of Human Chromosomes 21 and 22. Genome Research.

[B26] Ren B, Robert F, Wyrick JJ, Aparicio O, Jennings EG, Simon I, Zeitlinger J, Schreiber J, Hannett N, Kanin E, Volkert TL, Wilson CJ, Bell SP, Young RA (2000). Genome-wide location and function of DNA binding proteins. Science.

[B27] Iyer VR, Horak CE, Scafe CS, Botstein D, Snyder M, Brown PO (2001). Genomic binding sites of the yeast cell-cycle transcription factors SBF and MBF. Nature.

[B28] Horak CE, Snyder M (2002). ChIP-chip: a genomic approach for identifying transcription factor binding sites. Methods in Enzymology.

[B29] Oberley MJ, Tsao J, Yau P, Farnham PJ (2004). High-throughput screening of chromatin immunoprecipitates using CpG-island microarrays. Methods in Enzymology.

[B30] Weinmann AS, Yan PS, Oberley MJ, Huang H-MT, Farnham PJ (2002). Isolating human transcription factor targets by combining chromatin immunoprecipitation and CpG microarray analysis. Genes & Devel.

[B31] Cawley SE, Bekiranov S, Ng HH, Kapranov P, Sekinger EA, Kampa D, Piccolboni A, Sementchenko V, Cheng J, Williams AJ, Wheeler R, Wong B, Drenkow J, Yamanaka M, Patel S, Brubaker S, Tammana H, Helt G, Struhl K, Gingeras TR (2004). Unbiased mapping of transcription factor binding sites along human chromosomes 21 and 22 points to widespread regulation of noncoding RNAs. Cell.

[B32] Lieb JD, Liu X, Botstein D, Brown PO (2001). Promoter-specific binding of Rap1 revealed by genome-wide maps of protein-DNA association. Nat Genet.

[B33] Buck MJ, Lieb JD (2004). ChIP-chip: considerations for the design, analysis, and application of genome-wide chromatin immunoprecipitation experiments. Genomics.

[B34] Yang A, Zhu Z, Kapranov P, McKeon F, Church GM, Gingeras TR, Struhl K (2006). Relationships between p63 Binding, DNA Sequence, Transcription Activity, and Biological Function in Human Cells. Molecular Cell.

[B35] Fodor SP, Read JL, Pirrung MC, Stryer L, Lu AT, Solas D (1991). Light directed spatially addressable parallel chemical synthesis. Science.

[B36] Fodor SP, Rava RP, Huang XC, Pease AC, Holmes CP, Adams CL (1993). Multiplexed biochemical assays with biological chips. Nature.

[B37] Lipshutz R, Fodor SP, Gingeras TR, Lockhart D (1999). High density synthetic oligonucleotide arrays. Nat Genet.

[B38] Kapranov P, Sementchenko VI, Gingeras TR (2003). Beyond expression profiling: next generation uses of high density oligonucleotide arrays. Brief Funct Genomic Proteomic.

[B39] Mockler TC, Ecker JR (2005). Applications of DNA tiling arrays for whole-genome analysis. Genomics.

[B40] Bertone P, Gerstein M, Synder M (2005). Applications of DNA tiling arrays to experimental genome annotation and regulatory pathway discovery. Chromosome Research.

[B41] Bolstad B Probe Level Quantile Normalization of high Density Oligonucleotide Array Data. http://bmbolstad.com/stuff/qnorm.pdf.

[B42] Bolstad B, Irizarry R, Astrand M, Speed T (2003). Comparison of Normalization Methods for High Density Oligonucleotide Array Data Based on Bias and Variance. Bioinformatics.

[B43] Ghosh S, Hirsch H, Sekinger E, Struhl K, Gingeras T (2006). Rank-statistics based enrichment-site prediction algorithm developed for chromatin immunoprecipitation on chip experiments. BMC Bioinformatics.

[B44] Martone R, Euskirchen G, Bertone P, Hartman S, Royce TE, Luscombe NM, Rinn JL, Nelson FK, Miller P, Gerstein M, Weissman S, Snyder M (2003). Distribution of NF-kappaB-binding sites across human chromosome 22. PNAS.

[B45] Oppenheim AV, RW Schafer (1999). Discrete-time signal processing. Upper Saddle River (NJ): Prentice-Hall Inc.

[B46] Song JH, Kim JM, Kim SH, Kim HJ, Lee JJ, Sung MH, Hwang SY, Kim Tae Sung (2003). Comparison of the gene expression profiles of monocytic versus granulocytic lineages of HL-60 leukemia cell differentiation by DNA microarray analysis. Life Sciences.

[B47] Lee KH, Chang MY, Ahn JI, Yu DH, Jung SS, Choi JH, Noh YH, Lee YS, Ahn MJ (2002). Differential gene expression in retinoic acid-induced differentiation of acute promyelocytic leukemia cells, NB4 and HL-60 cells. Biochemical and Biophysical Research Communications.

[B48] Mattick J (2005). The Functional Genomics of Noncoding RNA. Science.

[B49] The FANTOM Consortium (2005). The transcriptional landscape of the mammalian genome. Science.

[B50] Bertone P, Stolc V, Royce TE, Rozowsky JS, Urban AE, Zhu X, Rinn JL, Tongprasit W, Samanta M, Weissman S, Gerstein M, Snyder M (2004). Global Identification of Human Transcribed Sequences with Genome Tiling Arrays. Science.

[B51] Wightman B, Burglin TR, Gatto J, Arasu P, Ruvkun G (1991). Negative regulatory sequences in the lin-14 3'-untranslated region are necessary to generate a temporal switch during Caenorhabditis elegans development. Genes Dev.

[B52] Integrated Genome Browser (IGB) is an open-source genome browser developed at Affymetrix. http://www.affymetrix.com/support/developer/downloads/TilingArrayTools/index.affx.

[B53] Larsson O, Wahlestedt C, Timmons JA (2005). Considerations when using the significance analysis of microarrays (SAM) algorithm. BMC Bioinformatics.

[B54] Lee TI, Rinaldi NJ, Robert F, Odom DT, Bar-Joseph Z, Gerber GK, Hannett NM, Harbison CT, Thompson CM, Simon I, Zeitlinger J, Jennings EG, Murray HL, Gordon DB, Ren B, Wyrick JJ, Tagne JB, Volkert TL, Fraenkel E, Gifford DK, Young RA (2002). Transcriptional Regulatory Networks in Saccharomyces Cerevisiae. Science.

[B55] R is a freely available language and environment for statistical computing. http://cran.rproject.org/.

